# Physiological, Nutritional and Metabolomic Responses of Tomato Plants After the Foliar Application of Amino Acids Aspartic Acid, Glutamic Acid and Alanine

**DOI:** 10.3389/fpls.2020.581234

**Published:** 2021-01-07

**Authors:** Marina Alfosea-Simón, Silvia Simón-Grao, Ernesto Alejandro Zavala-Gonzalez, Jose Maria Cámara-Zapata, Inmaculada Simón, Juan José Martínez-Nicolás, Vicente Lidón, Francisco García-Sánchez

**Affiliations:** ^1^Escuela Politécnica Superior de Orihuela, Universidad Miguel Hernández, Orihuela, Spain; ^2^Centro de Edafología y Biología Aplicada del Segura, Consejo Superior de Investigaciones Científicas, Murcia, Spain; ^3^Investigador Asociado al Departamento I+D Atlantica Agricola, Villena, Spain

**Keywords:** metabolites, nutrients, minerals, gas exchange parameters, ^1^H-NMR, organic acids, sugars

## Abstract

Agriculture is facing a great number of different pressures due to the increase in population and the greater amount of food it demands, the environmental impact due to the excessive use of conventional fertilizers, and climate change, which subjects the crops to extreme environmental conditions. One of the solutions to these problems could be the use of biostimulant products that are rich in amino acids (AAs), which substitute and/or complement conventional fertilizers and help plants adapt to climate change. To formulate these products, it is first necessary to understand the role of the application of AAs (individually or as a mixture) in the physiological and metabolic processes of crops. For this, research was conducted to assess the effects of the application of different amino acids (Aspartic acid (Asp), Glutamic acid (Glu), L-Alanine (Ala) and their mixtures Asp + Glu and Asp + Glu + Ala on tomato seedlings (*Solanum lycopersicum* L.). To understand the effect of these treatments, morphological, physiological, ionomic and metabolomic studies were performed. The results showed that the application of Asp + Glu increased the growth of the plants, while those plants that received Ala had a decreased dry biomass of the shoots. The greatest increase in the growth of the plants with Asp + Glu was related with the increase in the net CO_2_ assimilation, the increase of proline, isoleucine and glucose with respect to the rest of the treatments. These data allow us to conclude that there is a synergistic effect between Aspartic acid and Glutamic acid, and the amino acid Alanine produces phytotoxicity when applied at 15 mM. The application of this amino acid altered the synthesis of proline and the pentose-phosphate route, and increased GABA and trigonelline.

## Introduction

The tomato (*Solanum lycopersicum* L.) is one of the most-consumed and demanded crops worldwide due to its great nutritional and antioxidant properties. This high demand makes it one of the most important crops at the social and economic levels. In agreement with the statistics from the Food and Agriculture Organization [FAO] (2020), more than 180 million tons of tomato have been grown in the past year worldwide, of which 24 million come from Europe. Spain is the second-most important producer in Europe, with an approximate production of 5 million tons per year.

Due to the increase in the world’s population since the industrial revolution, the performance of the crops has had to increase fast and exponentially to provide and answer to the demand for food; however, in the past few years, the production and quality of the harvest of various crops, among which we find tomatoes, have been greatly affected as a result of climate change. The high temperatures and periods of drought, which have become more intense and prolonged, have affected crop production (Wallace et al., 2003; Parry, 2019; Mutale-Joan et al., 2020). Aside from the challenge faced by agriculture for increasing production in an era of climate change, we also find another important challenge, such as the practice of sustainable agriculture that affects the environment the least. Thus, at present, new agronomic strategies are being designed and evaluated, which could help take on these challenges, starting with the acquisition of basic scientific knowledge about the physiological and metabolic processes of crops. As an alternative to conventional agronomic strategies, such as the application of synthetic inorganic fertilizers, which could have damaging effects on the environment, other more ecological alternatives are being adopted (Foley et al., 2011; Tilman et al., 2002; Mutale-Joan et al., 2020). Among these new agronomic strategies, we find the use of biostimulants (Lucini et al., 2015). It has been observed that the use of these products significantly improves the performance of crops, as they have beneficial effects on the physiological processes of plants, such as the absorption of water and nutrients, among others (Mutale-Joan et al., 2020).

Biostimulants are composed of bioactive compounds such as humic acids, hydrolyzed proteins, seaweed and microorganism extracts, among others (Rouphael et al., 2018). The hydrolyzed proteins and seaweed extracts are a rich source of amino acids, and these compounds tend to be added freely to enrich the biostimulant products, as they have very beneficial effects on the crops due to their role in the synthesis of proteins, vitamins, nucleotides and alkaloids, aside from having effects as elicitors (Khan et al., 2019). Some of the most important effects described in the past few years are: (i) they palliate the negative impact of certain environmental stresses such as drought (L-methionine in bitter gourd, *Momordica charantia* L.; Akram et al., 2020), salinity (mixture of amino acids in tomato plants; Tantawy et al., 2009), Cd toxicity (Glutamate in rice; Jiang et al., 2020), (ii) they induce hormonal responses and improve the absorption of nitrogen (mixture of amino acids and polypeptides in tomato, corn and peas; Colla et al., 2014), (iii) they regulate the antioxidant metabolism (Glutamate, Cysteine, Phenylalanine, Glycine in soy Teixeira et al., 2017).

Despite the fact that as of today no doubts exist about the positive effects of the application of amino acids, as mentioned above, little is known about the role played by each of these amino acids on the physiological and metabolic processes of plants. On many occasions, the biostimulant products are formulated from the chemical hydrolysis of raw materials rich in amino acids (residues from the agro-food industry), but there is a lack of knowledge on their amino acid profile or what specific function they play in crops. Also, the application of biostimulant products rich in amino acids can vary depending on the species, cultivar, climate, dose used, origin and application time (Parađiković et al., 2019). Therefore, to create highly efficient biostimulant products, it is indispensable to understand what role the different amino acids play on the physiological and metabolic processes of crops, and the possible antagonistic, neutral and synergistic effects between them.

Amino acids such as Glutamic acid, Aspartic acid and Alanine are three of the amino acids used in the formulation of biostimulant products (Colla et al., 2015). These are added in their free form or as part of the raw materials from hydrolyzed proteins. **Glutamic acid** is important in the metabolism of nitrogen, as it intervenes in the assimilation of nitrogen in plants and in the reactions of amino transferases (Lea and Ireland, 1999; Cao et al., 2010). This amino acid, aside from its intrinsic value as an amino acid itself, is the precursor of other amino acids such as Aspartic acid, Serine, Alanine, Lysine and Proline, among others. **Alanine** is synthesized from Glutamic acid, and its transamination with oxoglutarate produces glutamate and pyruvate, a reversible reaction, granting this amino acid a dual function between carbon and nitrogen metabolism (Kendziorek et al., 2012). Lastly, this amino acid is linked with the synthesis of chlorophylls and photosynthetic activity (Sánchez-Pale, 2017). **Aspartic acid** is obtained from a transamination reaction between glutamate and oxaloacetate in plants, and is metabolized to produce the amino acids lysine, threonine, methionine, and isoleucine, in a series of reactions known as the Aspartic acid metabolic pathway. The application of these three amino acids to plants helps them to endure adverse environmental conditions such as drought, salinity and heavy metal toxicity (Rai, 2002). However, up to the present, the effects of the individual application of these amino acids on the physiology and the primary metabolism of plants, or the antagonistic or synergistic effects that could result from the mixture of amino acids, are unknown. This knowledge could be essential when designing biostimulants that contain amino acids, or even when selecting the raw materials for which the profile of amino acids is better adapted to the requirements of a specific crop. For this, the objectives of the study were to understand the effects of the application of the amino acids Glutamic acid, Aspartic acid and Alanine, applied individually or as a mixture, on the physiological and metabolic processes, and the nutritional state of tomato plants of the ‘Optima’ variety; and to discover if these changes stimulate the vegetative growth of the plants. To characterize the metabolic state of the plants, the ^1^H-NMR omics technique was utilized, as it allows analyzing a great quantity of primary metabolites in a fast and simple manner.

## Materials and Methods

### Growth Conditions and Plant Material

The experiment was performed on tomato plants (*Solanum lycopersicum* L.) of the ‘Optima’ variety, obtained from a commercial seedling nursery (Babyplant S.L., Santomera, Murcia, Spain), and planted in a tunnel greenhouse in an experimental field from the CEBAS-CSIC, located in the municipality of Santomera (18 Km from Murcia, Spain). The climatic conditions in the interior of the greenhouse were: temperature day/nigh 32/19 ± 3°C, photosynthetically active radiation (PAR) of 1000 μmol m^–2^ s^–1^, relative humidity of 52/80 ± 5% and a 15 h photoperiod.

The tomato seedlings, selected according to height and the number of completely developed true leaves, were transplanted to 3.5 L pots with a fine-grained “Universal” substrate, free of pathogens and weed seeds, composed of yellow and black peat, coconut fiber and perlite, in a ratio of 5:4:1 (Projar professional, 2018, Spain) The irrigation was applied through of dripper system of rate of 4 L h^–1^.

With regards to fertigation management, Hoagland’s nutrient solution, composed of KNO_3_ (27 g 100 L^–1^), Ca(NO_3_)_2_ (42 g 100 L^–1^), KH_2_PO_4_ (7 g 100 L^–1^), MgSO_4_ (13 g 100 L^–1^), Fe-EDTA (1 g 100 L^–1^), and micronutrients (1 g 100 L^–1^; Hidromix S, Valagro) was used in the irrigation. During the first week after the transplantation, plants were irrigated with 50% Hoagland solution one minute per day. After this week, plants were irrigated with 100% Hoagland solution two times per day, 5 min during the morning and 5 min during the afternoon. The tap water utilized for the preparation of the nutrient solution came from Tajo-Segura water transfer, with an electrical conductivity (EC) of 0.9 dS m^–1^ and pH of 8.2. When drainage in every irrigation event was less than 15% irrigation time was increased.

### Amino Acids Utilized and Preparation

The AAs utilized for the assays were Glutamic acid (Glu), Aspartic acid (Asp) and Alanine (L-Ala) from Caldic Ibérica (Barcelona, Spain). The selection of these three AAs and its doses was made according to previous experiments within the framework of project RTC-2016-4568-2 carried out at the CEBAS-CSIC. The aim of this project was to study every one of the 22 amino acids in different doses and mixtures in tomato plants. For each individual amino acid, a stock solution was first prepared with a final concentration of 7%. For this, 7 g of each amino acid were added to 100 mL of ultrapure water; in the case of Glutamic acid and Aspartic acid, drops of 50% KOH were added to facilitate their dilution in water. After the preparation of the individual AAs (7%), the following treatments were prepared: 15 mM Aspartic acid (Asp), 15 mM Glutamic acid (Glu), 15 mM Alanine (Ala), 15 mM Aspartic acid + 15 mM Glutamic acid (Asp + Glu) and 15 mM Aspartic acid + 15 mM Glutamic acid + 15 mM Alanine (Asp + Glu + Ala). For each formulation, before their application, Tween-20 was added to a concentration of 0.1% (a surfactant compound that improves the adherence of the formulation to the leaves), and the pH was adjusted to be 5.5 to 6.5. Therefore, the treatments applied were: (1) Control treatment (without AAs, in which only ultrapure water was pulverized on the tomato plants); and treatments with the foliar application of the amino acids: (2) Asp, (3) Glu, (4) L-Ala, (5) Asp + Glu, and (6) Asp + Glu + L-Ala. For each of the treatments, the concentrations of the equivalent nitrogen (mg L^–1^) were: Asp 0.22, Glu 0.20, L-Ala 0.33, Asp + Glu 0.42, and Asp + Glu + L-Ala 0.75.

### Application of the Treatments

The application of the treatments was performed foliarly, and was done after two weeks of acclimation of the tomato plants. Each treatment was pulverized on the aerial part of the plants, so that the greatest foliar area was covered with the preparation. Within the greenhouse, the plants were divided into four blocks, and within each block, the treatments were applied to four plants, which were considered as an experimental unit.

### Gas Exchange Parameters

After a week of treatment the following parameters were measured: the net CO_2_ assimilation rate (A_*CO*2_), stomatal conductance (g_*s*_), the water use efficiency (WUE = A_*CO*2_/E_*leaf*_, where E_*leaf*_ corresponds to the value of leaf transpiration obtained in each measurement), and the Ci/Ca ratio (where Ci corresponds to the substomatal CO_2_ and Ca corresponds to external CO_2_). These parameters were measured in two plants per treatment and block, in leaves that were completely developed and healthy, in the morning (8:30am - 11:00am, using a portable gas analyzer for the measurement of gas exchange parameters (PP System Ciras2, United Kingdom). During the measurements, the equipment was configured to maintain constant light (PAR: 1200 mmol m^–2^ s^–1^) and CO_2_ concentration (400 ppm) in the measurement chamber.

### Chlorophyll Fluorescence and Concentration Parameters

The chlorophyll fluorescence parameters were measured in the same plants utilized for the gas exchange measurements, utilizing a portable pulse-modulated fluorometer FMS-2 (Hansatech Instruments Ltd., United Kingdom). The chlorophyll fluorescence parameters measured were: the quantum efficiency of PSII, (ΦPSII = (F_*m*_′-F_*s*_)/F_*m*_′; the antennae efficiency of PSII, F_*v*_′/F_*m*_′ = (F_*m*_′-F_0_′)/F_*m*_′; and the photochemical quenching co-efficient, qP = (F_*m*_′-F_*s*_)/(F_*m*_′-F_0_′), where F_*s*_ is the steady-state fluorescence yield, F_*m*_′ is the maximal value when all reaction centers are closed after a pulse of saturating light (12,000 μmol m^–2^ s^–1^ for 0.8 s), and F_0_′ is the minimal fluorescence in the light-adapted state that is obtained by turning off the actinic light temporarily and applying a pulse of far-red light (735 nm) to drain the electrons from PSII. The chlorophylls were also measured with a portable meter CL-01 (SPAD units- Hansatech).

### Growth Parameters

After the gas exchange and chlorophyll fluorescence measurements, the following measurements were taken: height and diameter of the stem at three different points in all the plants. Also, the number of flowers of each plant and the percent of open flowers, after which the plants were harvested. In this sampling, the shoots of the plants were measured with a precision Sartorius scale (Acculab), after which they were dried in an oven at 60^*o*^C for at least 48 h (g dw shoot). From the shoots, eight leaflets were collected from each plant, which were washed with de-ionized water; four of them were dry-processed for the nutritional study, and the other four were processed fresh for the metabolomics study. The leaflets samples were taken from leaves that were completely developed, located mid-height of the plant.

### Determination of the Mineral Nutrients Concentration in Leaf Tissue

For the nutritional study, the previously dried and ground samples were analyzed. The concentration of Na, K, Mg, Ca, P, S, Fe, Cu, Mn, Zn and B was analyzed with inductively coupled plasma spectrometry (Iris Intrepid II, Thermo Electron Corporation, Franklin, United States), after digestion with HNO_3_:H_2_O_2_ (5:3 by volume), utilizing a microwave (CERM Mars Xpress, North Carolina, United States) with a temperature ramp that reached 200°C in 20 min, and maintaining this temperature for 2 h. The total N and C was measured with a C/N elemental analyzer Thermo Finnigan (Milan, Italy).

### Metabolic Analysis of Leaf Tissue

A “non-targeted” metabolic analysis was conducted in the fresh leaf samples. These samples were ground with liquid nitrogen with a mortar and pestle and lyophilized. Afterwards, the samples were prepared for analysis according with the protocol by Van der Sar et al. (2013). For this analysis, a Nuclear Magnetic Resonance (NMR) system coupled to a 500 MHz Bruker spectrometer (Bruker Biospin, Rheinstetten, Germany) equipped with a broadband 5 mm N_2_ CryoProbe Prodigy BBO. All the tomato leaf extracts were measured at 300.1 ± 0.1 K without rotation and with 4 test scans before the 32 scans performed for the experiment.

The acquisition parameters were set in the following manner: the size of the FID = 64K, spectral band = 12.4345 ppm, receiver gain = 28.5, acquisition time = 2.18 s, relaxation delay = 2s, and line broadening = 0.50 Hz. The acquisition of data was performed through the NOESY pulse sequence of pre-saturation (Bruker 1D, noesypr1d) with water suppression through the irradiation of the water frequency during the recycling and mixing times. In the procession of the samples and for each spectrum separately, a reduction of noise is produced, based on the deconvolution of the multi-level signal. Afterwards, a correction is performed of the baseline, and to complete the process, an interpolation technique of the areas of the signal is utilized.

All of this provides us with a “fingerprint” of the sample, a general view of the metabolites that are most represented produced by the cells at time of harvest, expressing the chemical shifts (δ) in parts per million (ppm). The NMR equipment detects the signals and records them as frequency vs intensity graphic, known as the “acquisition spectrum”.

The resulting ^1^H-NMR spectra were processed with the Chenomx NMR Suite program version 8.3 (Chenomx, Edmonton, Canada), in order to identify and quantify the metabolites of interest. All the samples were calibrated with the signal from the internal standard (IS), the deuterated Trimethylsilylpropionic acid sodium salt (TSP-d_4_) and the pH was set to a value of around 6. The software utilized includes a broad range of spectrum data which can be utilized to detect the metabolites that are over 5–10 μM: among the metabolites that were found and/or quantified, the following are highlighted: Aspartate, Glutamate, Alanine, Glutamine, Isoleucine, Valine, Tyrosine, Proline, Phenylalanine, Citrate, Formate, Fumarate, Malate, Fructose, Glucose, Sucrose, GABA, and Trigonelline (Duynhoven et al., 2013; Becerra-Martínez et al., 2017).

### Statistical Analysis

In this assay, the experimental design was one-factor (foliar application of the formulations) with a total of four experimental units (*n* = 4), which consisted of 4 biological units each. The statistical analysis included a one-way ANOVA with the SPSS program v24. When the ANOVA was significant (*p* < 0.05), Duncan’s HSD test for the separation of means was applied for *p* < 0.05. On the other hand, with the same statistics software, a Pearson’s correlation matrix was performed, which measured the degree of linear relationship between the variables. Also, to establish the existence or not of significant differences between the percent of metabolites compared with the control, a Student’s *t*-test was performed, for which the data were previously transformed through the arcsine of the root square of the value divided by one hundred.

A principal component analysis (PCA) and a cluster analysis (CA) were also performed. The cluster analysis was applied to the standardized data for hierarchical associations employing Ward’s method for agglomeration and the squared Euclidean distance as the dissimilarity measure.

## Results

### Vegetative Growth Parameters

**FIGURE 1:**
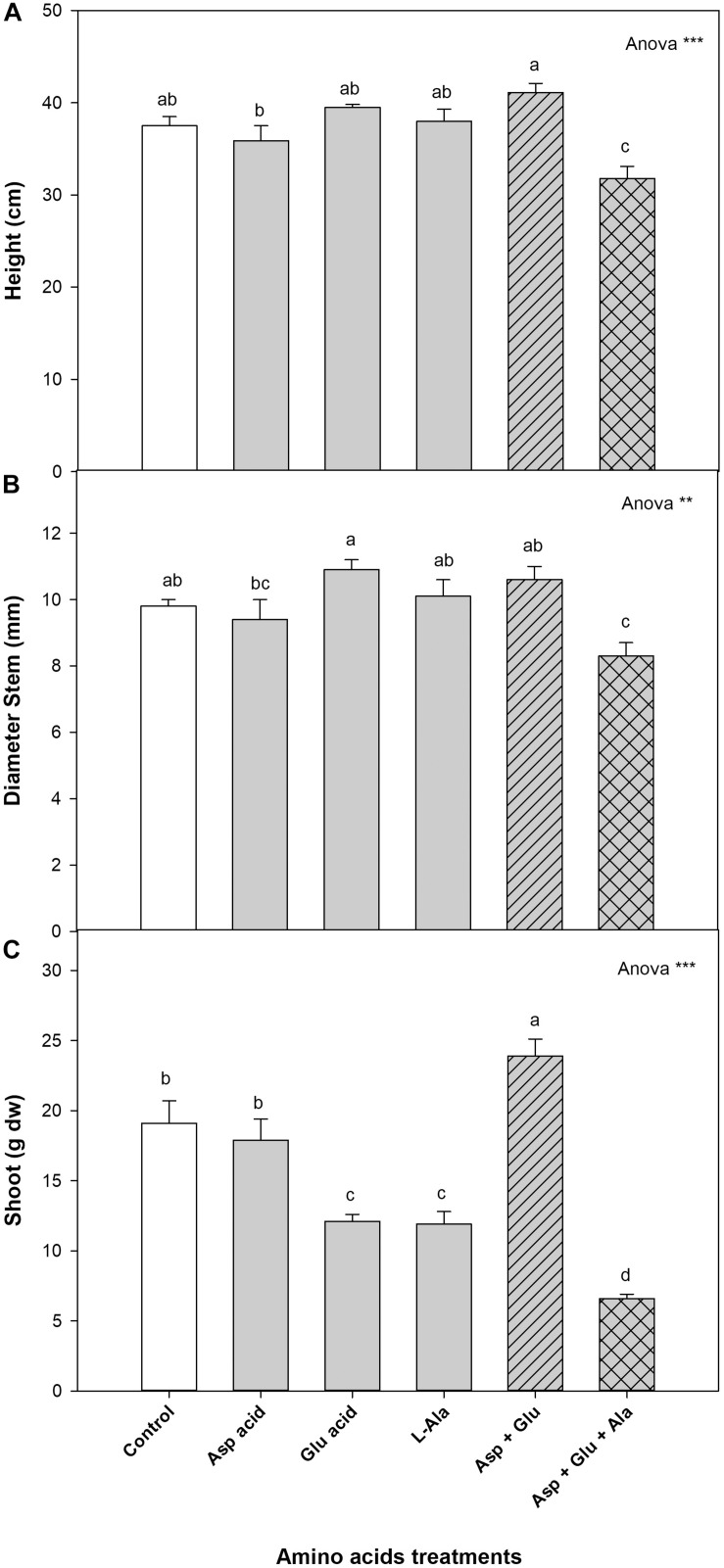
Growth parameters: height (cm) **(A)**, diameter stem (mm) **(B)** and shoot (g dw) **(C)**, measured in the ‘Optima’ variety of tomato after a week from the exogenous application of the treatment with AAs: Control (without AAs), Aspartic acid (Asp ac.), Glutamic acid (Glu ac.), L-Alanine (L-Ala), combination Asp + Glu and combination Asp + Glu + Ala. In the ANOVA: ** and *** indicate significant differences at *p* < 0.01 and *p* < 0.001, respectively. The different *lower case letters* indicate significant differences (*p* < 0.05) between the means established by Duncan’s test (*n* = 4).

The vegetative growth parameters were significantly affected by the treatments applied. Without the exogenous application of the AAs (control), the growth values were: a height of 37.5 cm, a stem diameter of 9.8 mm, and a dry weight of 19.1 g, respectively. The plants from the Asp + Glu treatment had a shoot dry weight that was significantly greater than the rest of the treatments, increasing by 25% with respect to the control plants. The plants with the least growth were those that were treated with the mixture of Asp + Glu + Ala, with a decrease in growth of 76%. The dry weight of the shoot decreased with the treatments in the following order: Asp + Glu > Control = Asp > Glu = Ala > Asp + Glu + Ala. As for the height, the plants treated with the Asp + Glu mixture were the tallest, but significant differences were only observed with the Asp and Asp + Glu + Ala treatments. The plants with the thickest stems were those that were treated with Glu, but significant differences were only observed with Asp and Asp + Glu + Ala (Figure 1).

### Physiological Study

**FIGURE 2:**
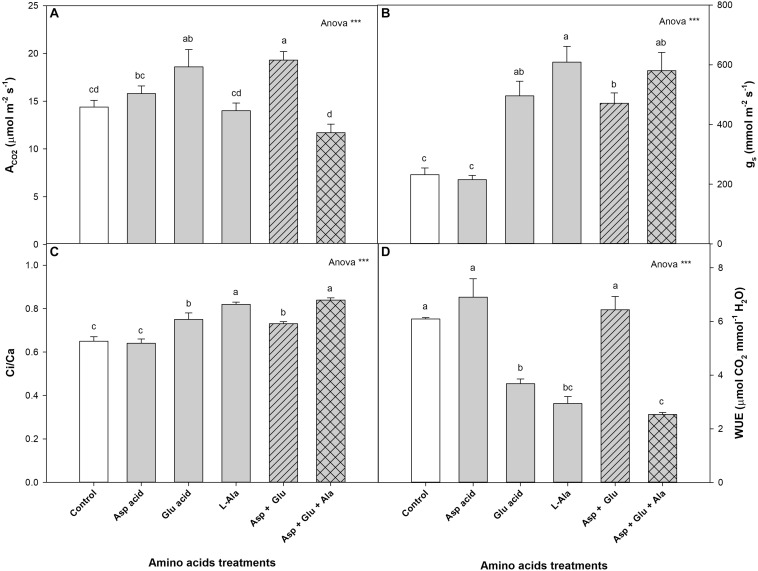
Gas exchange parameters: net CO_2_ assimilation rate (A_*CO*2_) **(A)**, stomatal conductance (g_*s*_) **(B)**, Ci/Ca ratio (Ci corresponds to the substomatal CO_2_ and Ca correspond to external CO_2_) **(C)** and water use efficiency (WUE) **(D)**, measured in the ‘Optima’ variety of tomato after a week from the exogenous application of the treatment with AAs: Control (without AAs), Aspartic acid (Asp ac.), Glutamic acid (Glu ac.), L-Alanine (L-Ala), combination Asp + Glu and combination Asp + Glu + Ala. In the ANOVA: *** indicate significant differences for *p* < 0.001. The different *lower case letters* indicate significant differences (*p* < 0.05) between the means established by Duncan’s test (*n* = 4).

For the parameters of gas exchange, it was observed that the plants without the exogenous application of AAs had the following values: A_*CO*2_ 14.4 μmol m^–2^ s^–1^, g_*s*_ 231.9 mmol m^–2^ s^–1^, Ci/Ca 0.65, and WUE 6.09 μmol mmol^–1^ (Figure 2). In all the parameters assayed, significant differences were observed with the treatments. The plants with the greatest net CO_2_ assimilation rate were those from the Asp + Glu treatment, although without significant differences found with respect to the Glu-treated plants. The lowest values were found in plants from the Asp + Glu + Ala treatments (although not significant with respect to the control and L-Ala). With respect to the g_*s*_ and Ci/Ca parameters, the control plants and those treated with Asp had values that were significantly lower than those from the rest of the treatments, while for WUE, the lowest values were found in plants treated with Glu, L-Ala and Asp + Glu + Ala.

The exogenous application of AAs did not affect neither the quantum efficiency of PSII (Φ_*PSII*_) nor the photochemical quenching co-efficient (qP). However, significant differences were observed between the treatments for the antennae efficiency parameter, in that the plants from the Asp treatment had the highest value, being significant with respect to those found in the Glu and Ala treatments.

**FIGURE 3:**
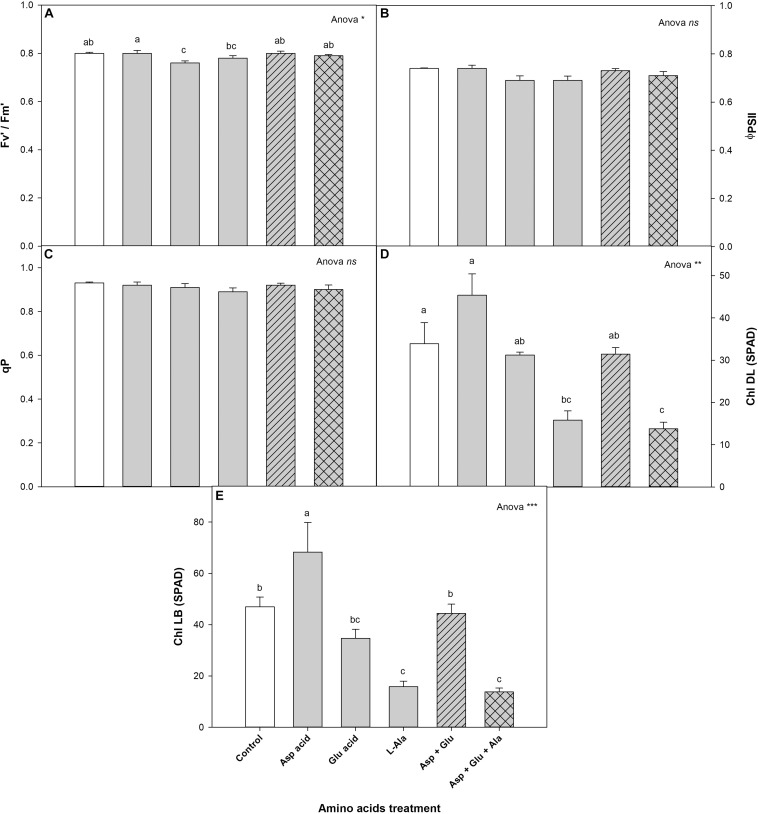
Chlorophyll fluorescence: antennae efficiency of PSII (Fv′/Fm′) **(A)**, quantum efficiency of PSII (Φ_*PSII*_) **(B)**, photochemical quenching co-efficient (qP) **(C)** and chlorophylls (Chl) measured in completely developed leaves (DL) **(D)** and leaf buds (LB) **(E)**, parameters measured in the ‘Optima’ variety of tomato after a week from the exogenous application of the treatment with AAs: Control (without AAs), Aspartic acid (Asp ac.), Glutamic acid (Glu ac.), L-Alanine (L-Ala), combination Asp + Glu and combination Asp + Glu + Ala. In the ANOVA: ‘*ns*’ indicates non-significant differences for a confidence interval of 95%; on their part, *, **, and *** indicate significant differences at *p* < 0.05, *p* < 0.01, and *p* < 0.001, respectively. The different *lower case letters* indicate significant differences (*p* < 0.05) between the means established by Duncan’s test (*n* = 4).

As for the concentration of chlorophylls, for the completely developed leaves (DL) as well as the buds (LB), significant differences were observed among the treatments (Figure 3). The control plants had chlorophyll values for DL and LB of 33.9 and 47.0 (SPAD values), respectively. The plants that were treated with Asp acid had the highest values of DL and LB, being significant for all the treatment in DL, and as compared to the control plants, and the Ala and Asp + Glu + Ala treatments for LB.

### Ionomic Study

For the plants that were not foliarly treated with AAs, the concentration of the macronutrients Ca, K, Mg, Na and P were 2.66, 2.95, 0.72, 0.19, 0.31, and 3.97 g 100 g^–1^ dw, respectively (Table 1). The concentration of Ca and Mg did not change due to the application of the AAs treatments with respect to the control treatment. However, significant differences were observed for K, P and N. These differences were due to the concentration of these nutrients increasing in the Ala and Asp + Glu + Ala treatments with respect to the other treatments with AAs and the control.

**TABLE 1:** Concentration of macro (g 100 g^–1^ dw) and micronutrients (ppm) quantified in leaves from the ‘Optima’ variety of tomato after a week from the exogenous application of the treatment with AAs: Control (without AAs), Aspartic acid (Asp ac.), Glutamic acid (Glu ac.), L-Alanine (L-Ala), combination Asp + Glu and combination Asp + Glu + Ala.

	Macronutrients (g 100 g^–1^ dw)				
Treatments	Ca	K	Mg	P	N
Control (without AAs)	2.66 ± 0.06	2.95 ± 0.04*cd*	0.72 ± 0.03	0.31 ± 0.02*bc*	3.97 ± 0.15b
Asp Ac.	2.73 ± 0.19	2.85 ± 0.19*cd*	0.67 ± 0.07	0.39 ± 0.04b	4.49 ± 0.43b
Glu Ac.	2.62 ± 0.20	2.72 ± 0.01d	0.71 ± 0.03	0.28 ± 0.01*c*	3.88 ± 0.27b
L-Ala	2.92 ± 0.23	4.50 ± 0.12b	0.66 ± 0.05	0.63 ± 0.04a	6.08 ± 0.24a
Asp + Glu	2.66 ± 0.21	3.33 ± 0.22*c*	0.71 ± 0.04	0.40 ± 0.04*b*	4.77 ± 0.17b
Asp + Glu + Ala	2.05 ± 0.16	5.42 ± 0.25a	0.64 ± 0.08	0.70 ± 0.02a	6.47 ± 0.30a
ANOVA	*ns*	***	*ns*	***	***

	**Micronutrients (ppm)**				
**Treatments**	**B**	**Cu**	**Fe**	**Mn**	**Zn**

Control (without AAs)	47.8 ± 1.3	8.7 ± 1.3b	171.2 ± 22.2a	71.7 ± 0.9*bc*	23.1 ± 0.7c
Asp Ac.	45.4 ± 1.2	11.8 ± 0.6b	121.9 ± 12.5*bc*	93.3 ± 2.3a	29.3 ± 1.7b
Glu Ac.	43.7 ± 0.7	9.3 ± 0.9b	104.7 ± 2.9c	84.8 ± 1.9*ab*	29.8 ± 1.9b
L-Ala	38.8 ± 2.8	11.3 ± 1.0b	125.5 ± 8.3*bc*	67.5 ± 2.6c	37.1 ± 2.7a
Asp + Glu	40.9 ± 4.7	8.9 ± 0.9b	105.0 ± 9.5c	76.6 ± 6.7*bc*	30.2 ± 3.1b
Asp + Glu + Ala	45.5 ± 5.2	22.7 ± 2.5a	154.2 ± 14.2*ab*	83.4 ± 6.5*ab*	36.4 ± 1.6a
ANOVA	*ns*	***	**	**	**

In the ANOVA: ‘ns’ indicates non-significant differences for a confidence interval of 95%; on their part, ** and *** indicate significant differences at p < 0.01 and p < 0.001, respectively. The different lower case letters indicate significant differences (p < 0.05) between the means established by Duncan’s test (n = 4).

The micronutrients results showed that for the control plants, the concentrations of B, Cu, Fe, Mn, and Zn were 47.8, 8.7, 171.2, 71.7, and 23.1 ppm, respectively (Table 1). In this case, there were significant differences in the concentrations of Cu, Fe, Mn and Zn. For Cu, these differences were due to the two-fold increase in concentration of the plants treated with Asp + Glu + Ala as compared to the rest of the treatments. The concentration of Fe oscillated between 171.2 ppm (control) and 104.85 (Glu and Asp + Glu), of Mn between 93.3 (Asp) and 67.5 (L-Ala), and of Zn between 36.8 (L-Ala and Asp + Glu + Ala) and 23.1 (Control), with significant differences observed between the maximum and minimum values.

### Metabolomic Study

#### Amino Acids

The AAs detected with ^1^H-NMR in tomato plants were Aspartate, Glutamate, Alanine, Tyrosine, Valine, Glutamine, Isoleucine, Phenylalanine and Proline. Of these AAs, in the control plants, the ones that added up to 98% of the total AAs detected were, in decreasing order, Glutamate (38%), Proline (30%), Glutamine (20%) and Aspartate (10%) (Table 2, Supplementary Figure 1). Of all the AAs measured, significant differences were observed with the treatments assayed, for Glutamate, Proline, Alanine, Tyrosine and Isoleucine. In the case of glutamate, it was observed that the application of L-Ala, Asp + Glu and Asp + Glu + Ala decreased its concentration with respect to the control, with the lowest values observed in plants treated with Asp + Glu + Ala. In the case of Proline, the treatments which included L-Ala (L-Ala and the Asp + Glu + Ala mixture) decreased its concentration, moving from 6.83 mg g^–1^ dw from the control plants to 2.5 mg g^–1^ dw, while the treatments Asp, Glu and their mixture (Asp + Glu) increased it, reaching the highest concentration in the mixture of 10.02 mg g^–1^ dw. The concentration of Alanine increased in the plants treated with this AA foliarly (L-Ala and Asp + Glu + Ala), and the Asp + Glu mixture, with the highest concentration measured in the plants treated with L-Ala. The concentration of Tyrosine decreased with the application of L-Ala and Asp + Glu + Ala. Likewise, the concentration of Isoleucine decreased with the application of Asp + Glu, in relation to the control plants (Table 2).

**TABLE 2:** Concentration of amino acids (mg g^–1^ dw) quantified by NMR in leaves from the ‘Optima’ variety of tomato after a week from the exogenous application of the treatment with AAs: Control (without AAs), Aspartic acid (Asp ac.), Glutamic acid (Glu ac.), L-Alanine (L-Ala), combination Asp + Glu and combination Asp + Glu + Ala.

	Amino Acids (mg g^–1^ dw)								
Treatments	Glutamate	Proline	Glutamine	Aspartate	Alanine	Phenylalanine	Valine	Tyrosine	Isoleucine
Control (without AAs)	7.85 ± 0.68a	6.83 ± 0.26c	4.68 ± 0.46	2.50 ± 0.26	0.60 ± 0.05*cd*	0.26 ± 0.03	0.25 ± 0.03	0.19 ± 0.03a	0.15 ± 0.03b
Asp Ac.	6.58 ± 0.42*abc*	8.63 ± 0.37b	4.89 ± 0.58	2.43 ± 0.23	0.56 ± 0.01*cd*	0.22 ± 0.03	0.24 ± 0.02	0.18 ± 0.04a	0.14 ± 0.03b
Glu Ac.	6.75 ± 0.70*ab*	8.13 ± 0.71b	4.02 ± 0.57	2.36 ± 0.23	0.48 ± 0.02d	0.30 ± 0.03	0.22 ± 0.03	0.15 ± 0.04*ab*	0.21 ± 0.04b
L-Ala	5.05 ± 0.19*cd*	2.47 ± 0.17d	3.15 ± 0.48	2.31 ± 0.16	1.44 ± 0.07a	0.18 ± 0.02	0.21 ± 0.02	0.06 ± 0.01b	0.20 ± 0.04b
Asp + Glu	5.54 ± 0.26*bcd*	10.02 ± 0.22a	2.82 ± 0.47	3.55 ± 0.42	0.66 ± 0.01c	0.27 ± 0.02	0.31 ± 0.03	0.13 ± 0.03*ab*	0.34 ± 0.04a
Asp + Glu + Ala	4.31 ± 0.43d	2.59 ± 0.04d	3.33 ± 0.40	2.53 ± 0.25	0.93 ± 0.03b	0.25 ± 0.02	0.21 ± 0.03	0.07 ± 0.01c	0.21 ± 0.04b
*ANOVA*	**	***	*ns*	*ns*	***	*ns*	*ns*	*	*

In the ANOVA: ‘ns’ indicates non-significant differences for a confidence interval of 95%; on their part, *, **, and *** indicate significant differences at p < 0.05, p < 0.01, and p < 0.001, respectively. The different lower case letters indicate significant differences (p < 0.05) between the means established by Duncan’s test (n = 4).

As for the relative distribution of the AAs, it was observed that in general, the treatments followed a model that was similar to the control plants, as previously commented, with some differences. The application of Asp, Glu and their mixture resulted in the % proline being higher than that of glutamate; and in the Asp + Glu mixture, the % of L-Ala overcame that of Glutamine (Supplementary Figure 1). On the other hand, the percentage of glutamine was higher than that of proline in the L-Ala and Asp + Glu + Ala treatments.

#### Organic Acids

**FIGURE 4:**
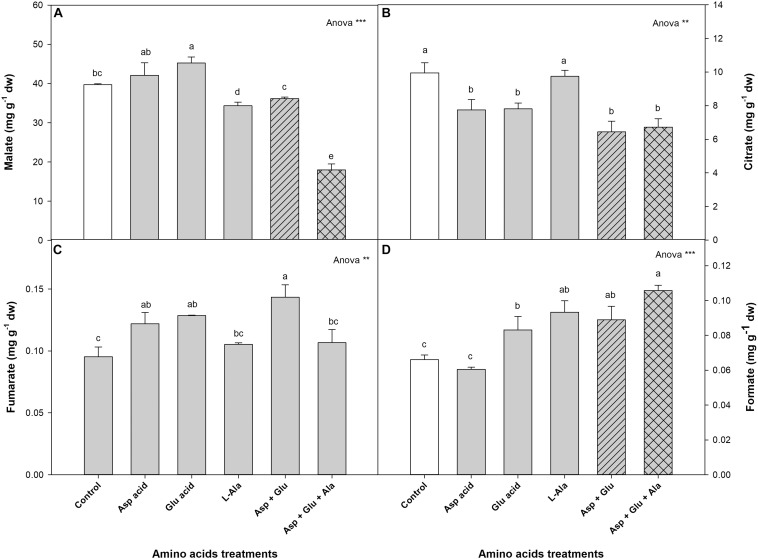
Concentration of organic acids (mg g^– 1^ dw): malate **(A)**, citrate **(B)**, fumarate **(C)**, and formate **(D)**, quantified by NMR in tomato leaves from the ‘Optima’ variety of tomato after a week from the exogenous application of the treatment with AAs: Control (without AAs), Aspartic acid (Asp ac.), Glutamic acid (Glu ac.), L-Alanine (L-Ala), combination Asp + Glu and combination Asp + Glu + Ala. In the ANOVA: ** and *** indicate significant differences for *p* < 0.01 and 0.001, respectively. The different *lower case letters* indicate significant differences (*p* < 0.05) between the means established by Ducan’s test. The vertical bar indicates the standard error of the mean (*n* = 4).

The organic acids quantified with ^1^H-NMR were malate, citrate, fumarate and formate (Figure 4, Supplementary Figure 2). Among these, the most common was malate, whose concentration comprised 80% of the total acids quantified, followed by citrate with 12%, with fumarate and formate being minor constituents. Thus, the concentration of malate increased with the application of Glu, and decreased in the plants from the L-Ala, Asp + Glu and Asp + Glu + Ala treatments, with respect to the control plants, reaching a concentration that was lower in the last treatment (Figure 4). The concentration of citrate decreased in all the treatments except for L-Ala, where no significant differences were found with respect to the control. The concentration of fumarate increased with the application of all the amino acids, with the highest value reached with the Asp + Glu mixture. The same trend was observed in the shoot concentration of formate, which increased in all the AAs treatments except for Asp. The greatest concentration was found for the Asp + Glu + Ala mixture, without significant differences observed with Asp + Glu and L-Ala. Despite the changes produced by the application of the AAs, the relative distribution of these in each treatment followed the same model as the one described previously for the control plants (Supplementary Figure 2).

#### Sugars

**FIGURE 5:**
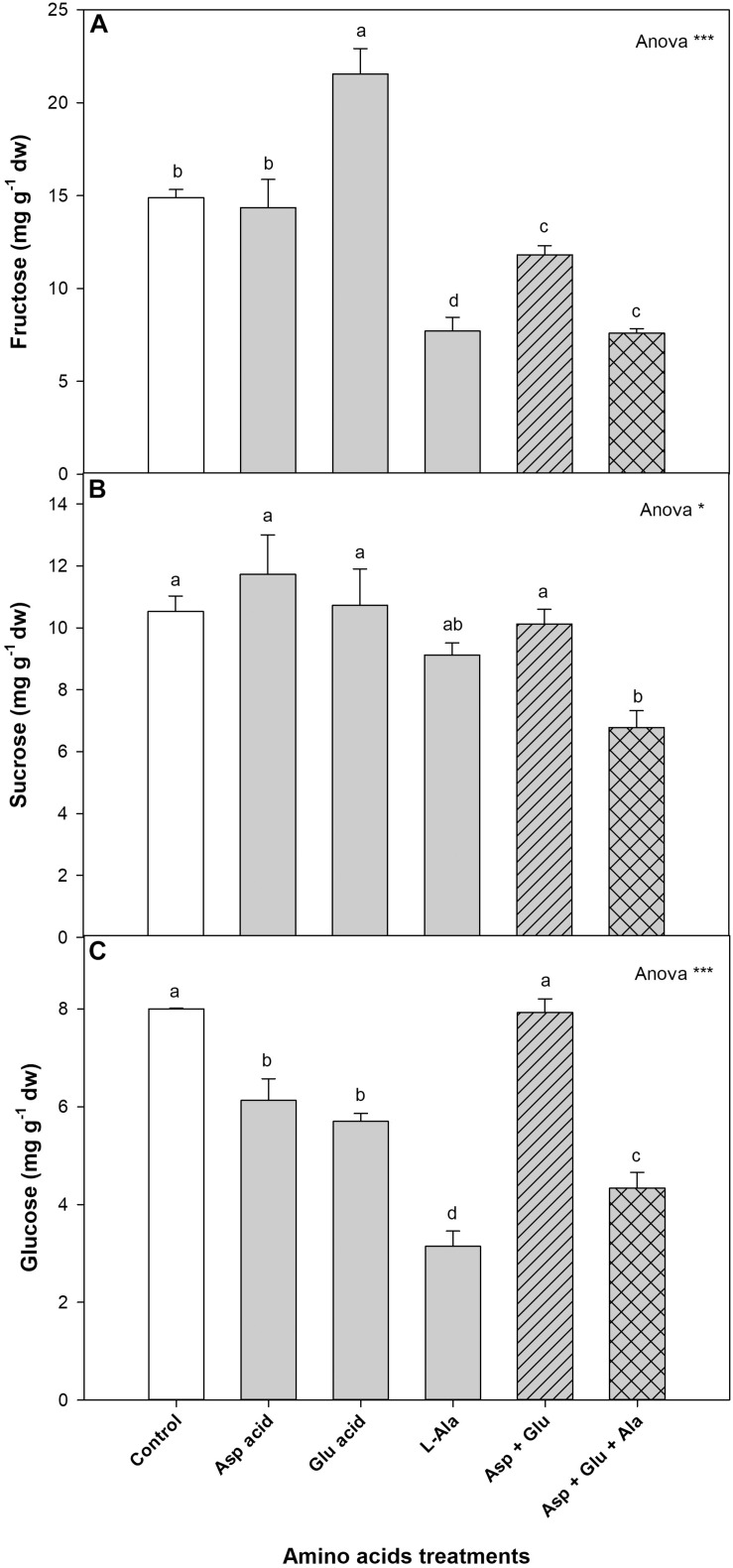
Concentration of sugars (mg g^– 1^ dw): fructose **(A)**, Sucrose **(B)**, and glucose **(C)**, quantified by NMR in tomato leaves from the ‘Optima’ variety of tomato after a week from the exogenous application of the treatment with AAs: Control (without AAs), Aspartic acid (Asp ac.), Glutamic acid (Glu ac.), L-Alanine (L-Ala), combination Asp + Glu and combination Asp + Glu + Ala. In the ANOVA: * and *** indicate significant differences for *p* < 0.05 and 0.001, respectively. The different *lower case letters* indicate significant differences (*p* < 0.05) between the means established by Ducan’s test. The vertical bar indicates the standard error of the mean (*n* = 4).

The sugars quantified with ^1^H-NMR in the leaves of the tomato plants were fructose, sucrose and glucose. Of these, the most commonly quantified was fructose (44.5%), followed by sucrose (31.5%) and glucose (24.0%) (Figure 5, Supplementary Figure 3). For all of them, it was observed that all the treatments affected their concentration. For fructose, the concentration increased with the application of Glu, while it decreased with the application of L-Ala and Asp + Glu + Ala, as compared to the control plants. For sucrose, the only significant difference observed between the treatments was the increase in its concentration in plants from the Asp treatment, as compared to the application of Asp + Glu + Ala. The concentration of glucose decreased with the application of Asp, Glu, L-Ala and Asp + Glu + Ala, with the L-Ala treatment having the lowest value. The relative distribution of these three sugars did not change in the AAs treatments with respect to the control treatment, as commented previously, except for the application of L-Ala, which resulted in a greater percentage of sucrose as compared to the percentage of fructose (Supplementary Figure 3).

#### Other Metabolites

**FIGURE 6:**
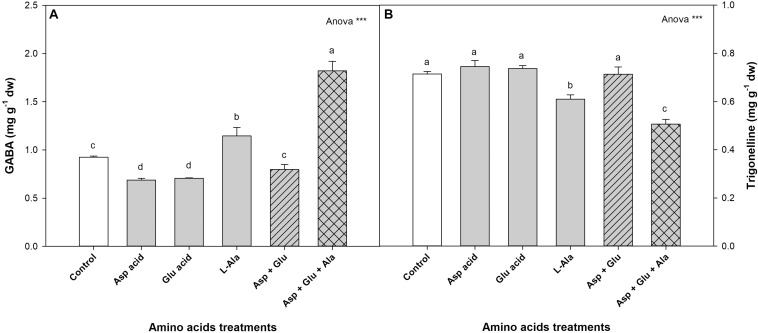
Concentration of GABA **(A)** and Trigonelline **(B)** (mg g^– 1^ dw) quantified by NMR in tomato leaves from the ‘Optima’ variety of tomato after a week from the exogenous application of the treatment with AAs: Control (without AAs), Aspartic acid (Asp ac.), Glutamic acid (Glu ac.), L-Alanine (L-Ala), combination Asp + Glu and combination Asp + Glu + Ala. In the ANOVA: *** indicates significant differences for *p* < 0.001. The different *lower case letters* indicate significant differences (*p* < 0.05) between the means established by Ducan’s test. The vertical bar indicates the standard error of the mean (*n* = *4*).

The ^1^H-NMR analysis also detected and quantified 4-Aminobutyrate (GABA) and Trigonelline (Figure 6). In normal conditions, without the application of AAs, the tomato plants have values of 0.92 mg g^–1^ dw for GABA and 0.71 mg g^–1^ dw for Trigonelline. In both cases, it was observed that the ANOVA was significant for the treatments. The application of the Asp + Glu + Ala mixture increased the concentration of GABA with respect to the rest of the treatments, increasing from 1.15 mg g^–1^ dw for the control treatment to 1.82 mg g^–1^ dw for this treatment. The concentration of Trigonelline decreased with the application of L-Ala and Asp + Glu + Ala, with the concentration being the lowest in the latter treatment.

### Principal Components Analysis (PCA) and Cluster Analysis

**FIGURE 7:**
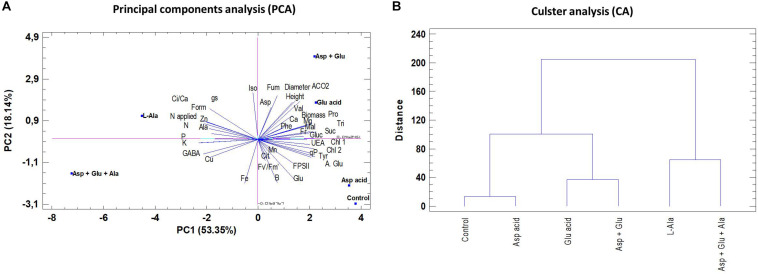
Principal component analysis (PC1 and PC2) **(A)** and Cluster analysis (CA) **(B)** in tomato leaves from the ‘Optima’ variety of tomato after a week from the exogenous application of the treatment with AAs: Control (without AAs), Aspartic acid (Asp ac.), Glutamic acid (Glu ac.), L-Alanine (L-Ala), combination Asp + Glu and combination Asp + Glu + Ala.

For a better and simpler visual interpretation of all the data, a principal component analysis and a cluster analysis were conducted (Figure 7). The first four components explained 93.75% of the variability, and the first three, represented by PC1, PC2 and PC3, explained 84%. The PC1 component explained 53.35% of the variability observed, thus showing that the variability was fundamentally due to WUE, qP, K, Mg, P, N, Glutamic acid, Proline, Tyrosine, Sucrose, GABA, Formate, and Trigonelline. The cluster analysis showed that L-Ala, and Asp + Glu + Ala treatments were more different to the rest of treatments.

## Discussion

Amino acids in plants are involved in primary and secondary metabolism, and participate in a wide range of cellular enzymatic reactions as constituents of different enzymes such as aminotransferases, dehydrogenases, lyases and decarboxylases. Therefore, they can have an influence on diverse phenological and physiological processes such as vegetative development of the plants, seed germination, fruit maturation, signaling and activation of defense systems against abiotic and biotic stresses, osmotic adjustment, deactivation of reactive oxygen species, and as a reserve source of nitrogen, among others (Teixeira et al., 2017). These important functions of the AAs in plants lead to the application of biostimulant products containing AAs, either added in their free form or added as botanical and seaweed extracts and hydrolyzed proteins, becoming a common practice in agriculture (Khan et al., 2019). Nevertheless, in order for the biostimulant products to become more efficient in the improvement of the agronomic performance of the crops, and so that they can be specifically formulated depending on the crop varieties, it necessary to understand the function of each amino acid in each crop, to identify antagonistic, synergistic or neutral effects among the amino acid mixtures, and to determine the doses and the best time of application.

In our experiment, it was observed that the foliar application of AAs can alter the vegetative growth of the plants. However, this effect was dependent on the AAs utilized for the preparation of the products applied. According to the shoot dry weight data (Figure 1), the best treatment found from all those applied was the Asp + Glu mixture. And, taking account that the application of these two AAs individually did not affect this parameter or even decreased it, as in the Glu treatment, relative to the control plants, it was concluded that there was a synergistic effect these two AAs. The opposite behavior was found for the plants to which L-Ala was applied individually or as part of a mixture. In this case, it became apparent that L-Ala was harmful to tomato plants when applied at a concentration of 15 mM. In addition, when mixed with Asp + Glu, its negative effect was enhanced, as observed in the decrease in dry weight of the shoot as well as the appearance of damage to the leaves (Figure 1). Some synergic effects between AAs has been previously reported. In an experiment conducted with this same tomato variety under saline conditions (Alfosea-Simón et al., 2020), it was observed that the Pro + Glu and Met + Trp mixtures were more efficient in palliating the effects of salinity as compared to the individual application of these AAs. Despite the positive effects of the application of Glutamic or Aspartic acids being observed on several occasions, such as in Kimchi plants grown in low temperatures (Lee et al., 2017) or rice under an excess of Cd (Rizwan et al., 2017), the positive effects of these two AAs applied simultaneously had never been previously described. On the other hand, plants from Asp + Glu + Ala applications received three times more amino acid content compared to those from individual Asp, Glu and Ala spraying, and 1.8 time more relative to Asp + Glu treatment. Thus, the excess of aminoacid content in the spraying Asp + Glu + Ala solution could have damaged the plants. In several cases, for instance, in Arabidopsis plants, it has been shown that amino acids as low as 100 μM applied via root caused damage in the root growth (Ravelo-Ortega et al., 2021). Thus, low concentrations of amino acids in some plant species can have un-expected effects.

**FIGURE 8:**
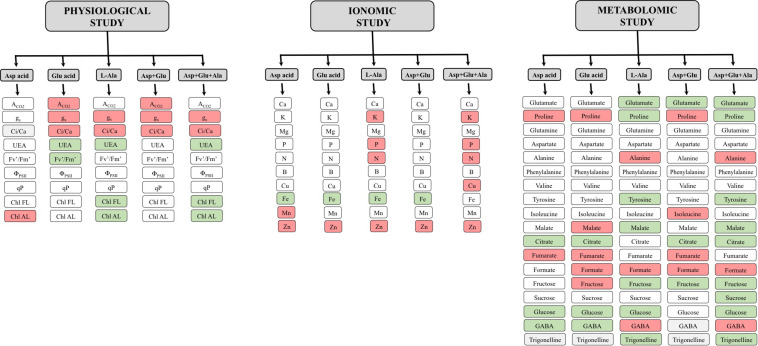
Summary of the relative results with respect to the control obtained after the foliar application of the AA treatments: Control (without AAs), Aspartic acid (Asp ac.), Glutamic acid (Glu ac.), L-Alanine (L-Ala), combination Asp + Glu and combination Asp + Glu + Ala. The red or green color indicate a significant increase or reduction, respectively, of the concentration of the metabolite in plants from the AA treatments compared with the control treatment. The white color indicates that no significant differences were found between the control and the corresponding AA treatment.

One of the reasons why the plants treated with Asp + Glu had a greater growth can be found in the results from the physiological study. In this study (Figure 8), it was observed that one of the common responses of the plants when treated with any AAs treatment was the increase in stomatal conductance (g_*s*_). However, this generalized response was not observed in the A_*CO*2_. The only plants that had an increase in the CO_2_ assimilation rate, in parallel to the opening of stomata, were those from the Asp + Glu treatment. Thus, the simultaneous effect of Asp + Glu could have enhanced the biochemical reactions of photosynthesis. This has already been reported by Lee et al. (2017) with the foliar application of Glutamic acid, with positive effects observed on the photosynthetic rate and stomatal conductance. In an experiment carried out in rice plants under conditions of cadmium stress, it was determined that the foliar application of Asp also improved the photosynthetic rate and stomatal conductance (Rizwan et al., 2017). In our experiment in tomato plants, the simultaneous application of Asp + Glu was necessary to enhance A_*CO*2_. In addition, in these plants, A_*CO*2_ and g_*s*_ had similar increases (as a percentage), relative to control plants. Thus, the WUE was maintained similar to the control plants, resulting in the maintenance of a good balance between the CO_2_ captured and water lost by the plants. On the other hand, the application of L-Ala seems to have altered the non-stomatal factors of photosynthesis (biochemical factors), as indicated by the decrease in the assimilation rate of CO_2_ in plants treated with L-Ala and Asp + Glu + Ala in parallel to the decrease in the Ci/Ca ratio (Farquhar and Sharkey, 1982). In plant cell culture studies, evidence has been shown about the harmful effects of L-Alanine. Chen et al. (2006) in cell cultures of *V. labrusca* and *V. vinifera* observed that the application of L-Ala to the growth media caused the death of cells due to the activation of Phenylalanine ammonium lyase, 4-hydroxylase cinnamic acid, the expression of the stilbene synthase gene, and the accumulation of stilbenes. Nevertheless, the applications of L-Ala to crops has barely been studied, except in the *Plumbago indica* crop, where the application of Alanine (5mM) combined with chitosan (150 mg L^–1^) improved its production (Jaisi and Panichayupakaranant, 2020).

The ionomics study indicated that the AAs applied to the tomato plants of the variety ‘Optima’ did not induce changes in the absorption of nutrients relative to control plants, as the concentrations of macronutrients and micronutrients in plants from any of the treatments were within the normal range for this crop (Watanabe et al., 2016). However, some foliar treatments with amino acids are able to increase or decrease the absorption of diverse macronutrients and micronutrients as indicated by Sainju et al. (2003). The data from our experiment showed that the plants treated with L-Ala (L-Ala and Asp + Glu + Ala) had an altered nutritional status as in these plants it was increased the K, P and N concentration. This could be due to a concentration effect, as the dry weight of the shoot decreased. However, as this effect was not observed in the concentration of Ca and Mg, it should not be discarded that the L-Ala amino acid could have influenced the absorption and transport of the nutrients in plants. Nevertheless, the increase in K, P and N with respect to Ca and Mg could have created nutritional imbalances which may have caused a decrease in the growth of the shoots. Another possible reason for the highest leaf N concentration observed in the Asp + Glu + Ala treated plants could be due to the high N concentration (0.75 mg N L^–1^) of the solution sprayed relative to the rest of treatments.

In the metabolomics study, it was observed that the most significant change observed after the application of the AAs Aspartic acid, Glutamic acid and their Asp + Glu mixture, occurred in the concentration of Proline. The Asp and Glu treatments, and their Asp + Glu mixture increased the concentration of this amino acid, and this increase was greater with the Asp + Glu mixture. Proline is an AA with multiple functions. It has the ability to act as an osmoprotector, and plays a role in osmotic adjustment, deactivation of free oxygen radicals, regulation of the absorption of nutrients, and can also induce the CO_2_ assimilation rate (Pervaiz et al., 2019). These functions are especially important when the plants suffer adverse environmental conditions. In the Mediterranean area, the typical greenhouse conditions can make temperature be a limiting factor in tomato cultivation. Therefore, the increase of proline in these plants could have exerted a protective role that favored the increase in the CO_2_ assimilation rate and the plant’s growth. In addition, glutamate, aside from being a precursor of proline, is also related to the production of glutathione and arginine. These two compounds are involved in oxidative metabolism, directly as glutathione or indirectly as arginine, as they activate PAL activity (Ben-Rejeb et al., 2014; Zheng et al., 2011). On the contrary, the application of L-Ala and Asp + Glu + Ala decreased the concentration of proline, and this response coincided with a decrease of A_*CO*2_ and decreased shoot growth. These results suggest that Asp + Glu improved the physiological and morphological parameters of the plants through the synthesis of proline, while Ala altered the metabolism of the plants by reducing the concentration of proline, and therefore reducing their growth.

In plants, the AAs Glutamate and Aspartate are precursors of up to eleven AAs. The reaction of Glutamate + Oxaloacetate produces Aspartate + α-ketoglutarate. This reaction is reversible, so that in the biosynthesis pathways, the AAs from glutamate (such as Alanine, Valine, Leucine, Glutamine, Proline and Arginine) are connected with those from Aspartate (such as Asparagine, Methionine, Threonine, Isoleucine and Lysine) (Forde and Lea, 2007). The application of Glutamate and Aspartate and their mixture did not increase the foliar concentration of these AAs, but were metabolized to increase the concentration of proline. Instead, the application of L-Ala individually or in a mixture increased the concentration of L-Ala in leaves. This AA was not metabolized, so that the increase in concentration could have caused damages to the cells, as observed in Chen et al. (2006) in cell cultures. Another of the alterations produced by the application of L-Ala was the decrease in Tyrosine. This AA is the precursor of secondary metabolites derived from the p-Coumaroyl-CoA pathway, which produces compounds such as coumarins, flavonoids, isoflavonoids, stilbenes, aurones, cutin, suberin, sporopollenin, catechins, proanthocyanidins, lignans, lignins, phenylpropenes, acylated polyamines and many other alkaloid derivatives. These compounds are related with the antioxidant protection capacity of the cells and with the mechanisms of protection of plants against plagues and diseases.

In the metabolomics study it was also observed that some compounds from the pentose phosphate pathway, such as fructose, sucrose, glucose and formate, as well as the tricarboxylic acid pathway (citrate, malate and fumarate), were affected. For the individual application of Asp, Glu and L-Ala and their mixture Asp + Glu, different responses were observed in the pentose phosphate pathway and the tricarboxylic acid one, which demonstrate that the mix of amino acids has a different effect than the AA applied individually. The mixtures that contained L-Ala produced a decrease in the concentration of fructose, sucrose and glucose, malate and citrate. This could suggest that the L-Ala applied at a concentration of 15mM produces alterations in the metabolism of C such as in A_*CO*2_, which could have caused a decrease in the growth of the shoot. Therefore, the causes of L-Ala toxicity in tomato plants should be studied further, using as the report from Chen et al. (2006) a reference, where it was observed that AAs can activate the enzymes Phenylalanine ammonium lyase, 4-hydroxylase cinnamic acid, the expression of the stilbene synthase gene, and the accumulation of stilbenes. In the case of Asp, Glu, and their mixture, it was observed that while the mixture maintained the concentration of glucose at similar values as those of the control, the individual application decreased its concentration. This could indicate that the simultaneous application of Asp + Glu improved the capture and assimilation of CO_2_ in the plants, improving the growth of these plants, where proline could have played an important role in increasing the performance of the photosynthetic system. For the tricarboxylic acid pathway, it was observed that the application of this Asp + Glu treatment induced a reduction in the concentration of citrate, and an increase in the concentration of fumarate, which could have modulated the A_*CO*2_ and g_*s*_ regulation in plants under this treatment. Some studies have revealed a negative correlation between the concentrations of fumarate and gas exchange through the opening of stomata (Nunes-Nesi et al., 2007; Araújo et al., 2012).

Another of the responses that differentiated the treatments that contained L-Ala with those that did not have this AA, was observed in the concentrations of GABA and Trigonelline. The GABA increased while the Trigonelline decreased with respect to the control, and this response was greater when L-Ala was applied with the Asp + Glu mixture. GABA is a non-protein amino acid with multiple functions in plants, playing an important role in metabolic processes such as signaling, interconnection between Carbon and Nitrogen metabolisms and the tolerance to different stresses such as low light intensity, salinity, and lack of nitrogen, drought or temperature (Ramos-Ruiz et al., 2019). The increase in GABA observed in plants treated with L-Ala could be the result of some type of stress produced by this amino acid, unchaining a protection response related with GABA. In these plants, this response was not enough to counter the stress created, and therefore, led to a decrease in growth. The concentration of Trigonelline also increases in the plant as a response to different stresses, mainly salinity, drought or UV light (Cho et al., 1999; Cho et al., 2003; Willmon et al., 2017). However, the application of L-Ala in this experiment decreased its concentration. It is important to point out that the increased in GABA and the decrease of Trigonelline are found in plants suffering some abiotic stress, so that these metabolites could be used as biomarkers to detect the possible negative effects of these AAs in tomato plants. However, more research is needed to verify this hypothesis.

The different changes observed in the metabolic processes of the plants utilized in the present research could be due that the AAs, aside from having physiological functions, may also be involved in the metabolism of plants in signaling processes (Häusler et al., 2014). It has been described that plants have glutamate receptors (GLRs) that could be activated not only by glutamate, but also by other AAs such as Serine, Alanine, Methionine, Tryptophan, Phenylalanine, Leucine, Asparagine, Threonine, Cysteine, Glycine, Tyrosine, and Peptides (Vincill et al., 2012; Forde and Roberts, 2014). The activation of these receptors could unchain a series of signaling mechanisms in processes related to the absorption of nitrogen by the roots (Miller et al., 2008), growth and architecture of the roots (Weiland et al., 2016), antioxidant metabolism (Hildebrandt et al., 2015; Weiland et al., 2016), regulation of transcription factors (Santi et al., 2017), regulation of stomas and photosynthesis, and plant defense (Weiland et al., 2016), as well as balances between C and N metabolism (Kang and Turano, 2003; Price et al., 2012).

## Conclusion

In this experiment, it was observed that the application of AA to tomato plants could be beneficial for increasing their vegetative development, but this application should be first optimized. To increase the efficiency of biostimulant products in agriculture, much research is needed that may reveal: i) the effects of the AAs on physiological and metabolic processes of plants, either applied individually or as a mixture; in this experiment it was observed that the combined application of the AAs (in this case Asp and Glu) had better effects that their individual application, and ii) what dose needs to be applied; in this experiment, it was observed that the application of 15 mM of Alanine is toxic to tomato plants, and it is not reversed by the simultaneous application of the Asp + Glu + Ala mixture. The application of AAs to plants resulted in changes in their ionomic, physiological and metabolomic profiles; however, as these compounds have many different functions in plants, a systems biology approach that integrates metabolomics, proteomic, genomic and hormonal studies to delve into these changes, is necessary.

## Data Availability Statement

The raw data supporting the conclusions of this article will be made available by the authors, without undue reservation.

## Author Contributions

FG-S and EZ-G conceived and designed the research. MA-S, SS-G, IS, and VL conducted the experiments and collected the data in the field and in the lab. JC-Z and JM-N analyzed the data and did tables and figures. FG-S and MA-S wrote the manuscript. SS-G and EZ-G commented and revised the manuscript. All authors contributed to the article and approved the submitted version.

## Conflict of Interest

EZ-G was employed by the company Atlantica Agricola. The remaining authors declare that the research was conducted in the absence of any commercial or financial relationships that could be construed as a potential conflict of interest.

## Supplementary Material

Supplementary Figure 1 Profile of the concentration of AAs (%) quantified with Nmr in tomato plant leaves from the ‘Optima’ variety after a week from the exogenous application of the treatment with AAs: Control (without AAs), Aspartic acid (Asp ac.), Glutamic acid (Glu ac.), L-Alanine (L-Ala), combination Asp + Glu and combination Asp + Glu + Ala. ^∗^ indicates significant differences at *p* < 0.05 between the means established by Student’s *t-test* (*n = 4*).

Supplementary Figure 2 Profile of the concentration of organic acids (%) quantified by RMN in tomato leaves from the ‘Optima’ variety of tomato after a week from the exogenous application of the treatment with AAs: Control (without AAs), Aspartic acid (Asp ac.), Glutamic acid (Glu ac.), L-Alanine (L-Ala), combination Asp + Glu and combination Asp + Glu + Ala. ^∗^ indicates significant differences at *p* < 0.05 between the means established by Student’s *t-test* (*n = 4*).

Supplementary Figure 3 Profile of the concentration of sugars (%) quantified by Nmr in tomato leaves from the ‘Optima’ variety of tomato after a week from the exogenous application of the treatment with AAs: Control (without AAs), Aspartic acid (Asp ac.), Glutamic acid (Glu ac.), L-Alanine (L-Ala), combination Asp + Glu and combination Asp + Glu + Ala. ^∗^ indicates significant differences at *p* < 0.05 between the means established by Student’s *t-test* (*n = 4*).
