# Supplementary figures and images for: Physiological, Nutritional and Metabolomic Responses of Tomato Plants After the Foliar Application of Amino Acids Aspartic Acid, Glutamic Acid and Alanine
Source: Front Plant Sci. 2021 Jan 7;11:581234. doi: 10.3389/fpls.2020.581234 (PMC7817619; doi:10.3389/fpls.2020.581234)

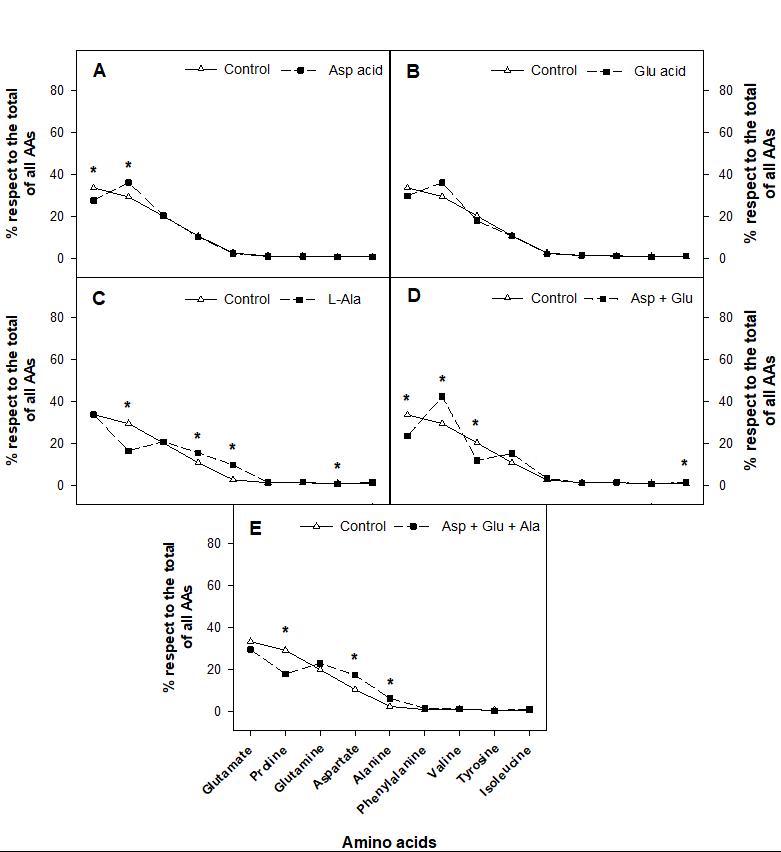

Supplement: Supplementary Figure 1 — Profile of the concentration of AAs (%) quantified with Nmr in tomato plant leaves from the ‘Optima’ variety after a week from the exogenous application of the treatment with AAs: Control (without AAs), Aspartic acid (Asp ac.), Glutamic acid (Glu ac.), L-Alanine (L-Ala), combination Asp + Glu and combination Asp + Glu + Ala. ∗ indicates significant differences at p < 0.05 between the means established by Student’s t-test (n = 4). [file Image_1.JPEG]

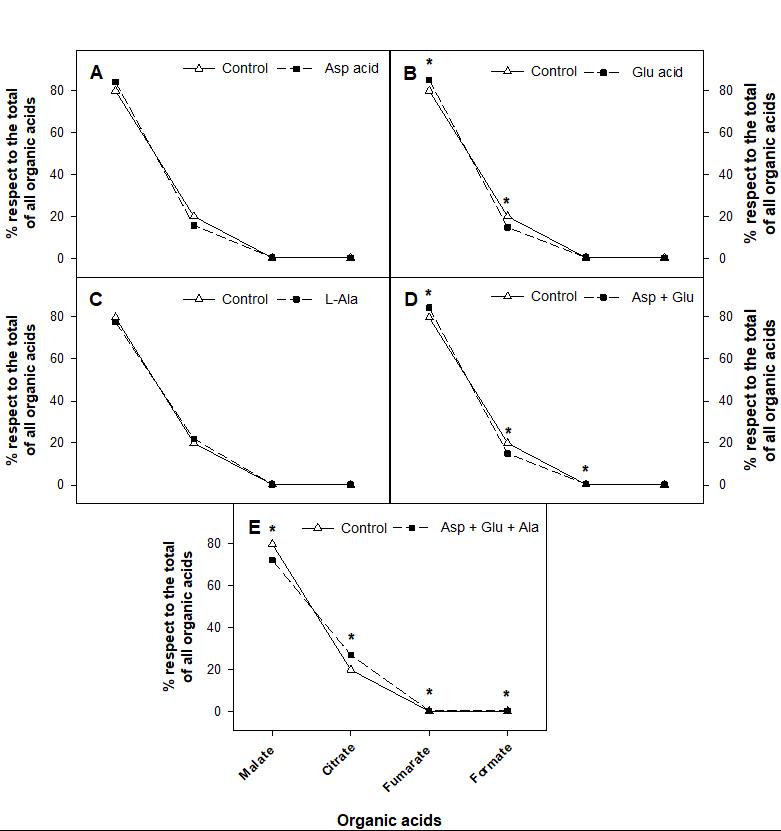

Supplement: Supplementary Figure 2 — Profile of the concentration of organic acids (%) quantified by RMN in tomato leaves from the ‘Optima’ variety of tomato after a week from the exogenous application of the treatment with AAs: Control (without AAs), Aspartic acid (Asp ac.), Glutamic acid (Glu ac.), L-Alanine (L-Ala), combination Asp + Glu and combination Asp + Glu + Ala. ∗ indicates significant differences at p < 0.05 between the means established by Student’s t-test (n = 4). [file Image_2.JPEG]

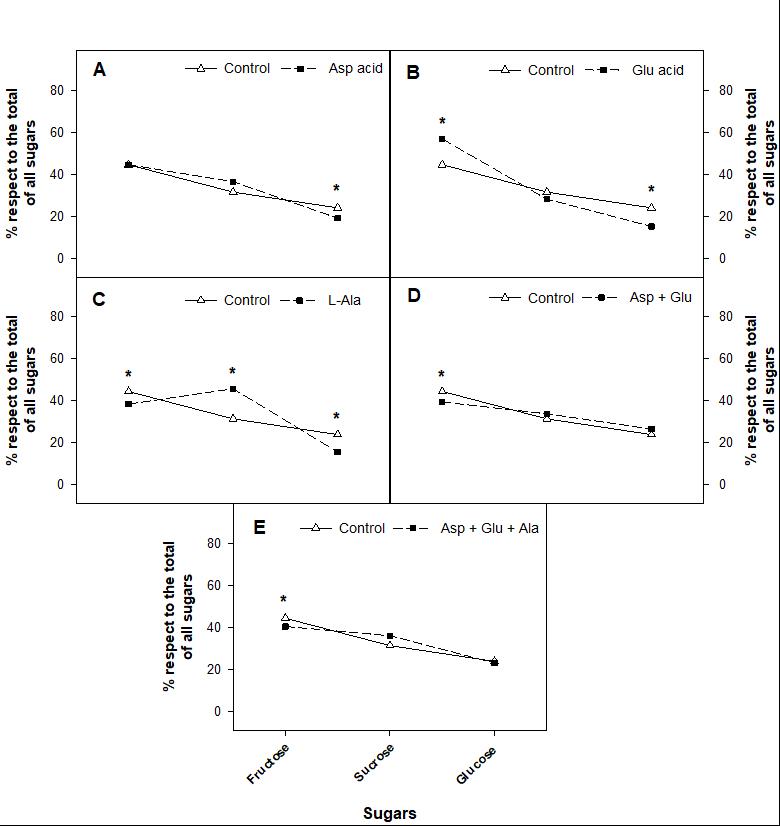

Supplement: Supplementary Figure 3 — Profile of the concentration of sugars (%) quantified by Nmr in tomato leaves from the ‘Optima’ variety of tomato after a week from the exogenous application of the treatment with AAs: Control (without AAs), Aspartic acid (Asp ac.), Glutamic acid (Glu ac.), L-Alanine (L-Ala), combination Asp + Glu and combination Asp + Glu + Ala. ∗ indicates significant differences at p < 0.05 between the means established by Student’s t-test (n = 4). [file Image_3.JPEG]
